# Invasive stratified mucin-producing carcinoma of the uterine cervix masquerading as squamous cell carcinoma on biopsy: a diagnostic pitfall

**DOI:** 10.1093/omcr/omag087

**Published:** 2026-06-08

**Authors:** Yoichiro Okubo, Shuka Wada, Shu Yuguchi, Tomomi Yokozawa, Haruya Saji, Yohei Miyagi

**Affiliations:** Department of Pathology, Kanagawa Cancer Center, 2-3-2 Nakao, Asahi-ku, Yokohama, Kanagawa 241-8515, Japan; Department of Pathology, Kanagawa Cancer Center, 2-3-2 Nakao, Asahi-ku, Yokohama, Kanagawa 241-8515, Japan; Department of Pathology, Japanese Red Cross Medical Center, 4-1-22 Hiroo, Shibuya-ku, Tokyo 150-8935, Japan; Department of Pathology, Kanagawa Cancer Center, 2-3-2 Nakao, Asahi-ku, Yokohama, Kanagawa 241-8515, Japan; Department of Gynecology, Kanagawa Cancer Center, 2-3-2 Nakao, Asahi-ku, Yokohama, Kanagawa 241-8515, Japan; Department of Gynecology, Kanagawa Cancer Center, 2-3-2 Nakao, Asahi-ku, Yokohama, Kanagawa 241-8515, Japan; Department of Pathology, Kanagawa Cancer Center, 2-3-2 Nakao, Asahi-ku, Yokohama, Kanagawa 241-8515, Japan; Molecular Pathology and Genetics Division, Kanagawa Cancer Center Research Institute, 2-3-2 Nakao, Asahi-ku, Yokohama, Kanagawa 241-8515, Japan

**Keywords:** invasive stratified mucin-producing carcinoma, stratified mucin-producing intraepithelial lesion, uterine cervix, squamous cell carcinoma, p40, mucin stain

## Abstract

Invasive stratified mucin-producing carcinoma (ISMC) of the uterine cervix can masquerade as non-keratinizing squamous cell carcinoma (SCC) on small biopsies because it often grows as solid nests with minimal gland formation and little or no keratinization, and may show patchy cytokeratin 5/6 (CK5/6) positivity. We report a 44-year-old woman whose outside cervical biopsy was interpreted as SCC. Repeat biopsy showed SCC-like solid nests without keratinization and patchy CK5/6 positivity, but p40 and p63 were negative and periodic acid-Schiff with diastase (D-PAS) demonstrated diastase-resistant intracytoplasmic mucin within tumor nests. Radical hysterectomy revealed a human papillomavirus (HPV)-associated endocervical adenocarcinoma with a predominant ISMC component, lymphovascular invasion, and nodal metastases. This case highlights a practical diagnostic pitfall and supports a minimal, reproducible workup for p16 block-positive, SCC-like biopsies: p40/p63 plus a mucin stain (D-PAS and/or Alcian blue), with PAX8 as needed. Funding: Japan Society for the Promotion of Science (JSPS) KAKENHI [25 K10294].

## Graphical Abstract

**Graphical Abstract:**
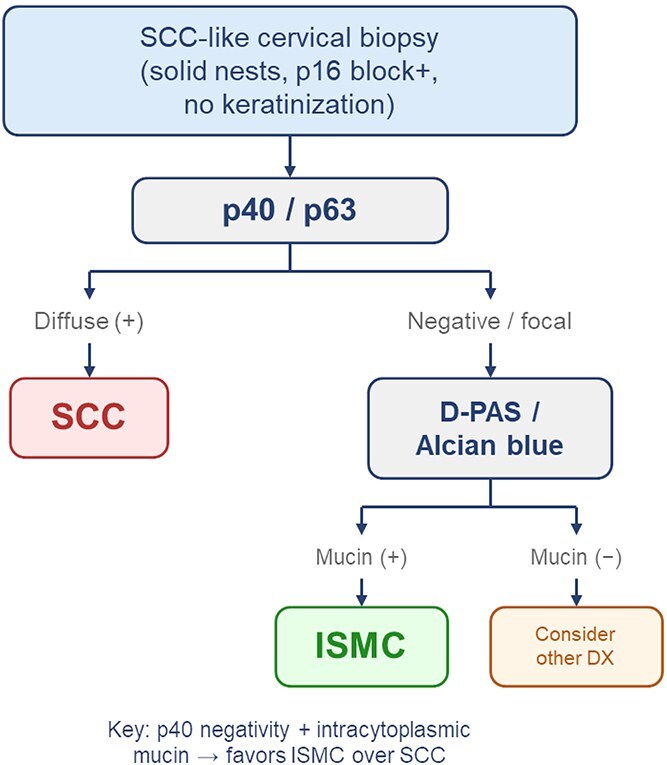

## Introduction

Stratified mucin-producing intraepithelial lesion (SMILE) and its invasive counterpart, invasive stratified mucin-producing carcinoma (ISMC), are uncommon but increasingly recognized within the spectrum of human papillomavirus (HPV)-associated endocervical adenocarcinoma [1–3]. ISMC often grows as solid nests with minimal gland formation and little or no keratinization, creating a practical diagnostic pitfall in small biopsies where the tumor may be mistaken for non-keratinizing squamous cell carcinoma (SCC) [2, 4, 5]. Compounding this, cytokeratin 5/6 (CK5/6) can be patchy to diffuse in HPV-associated endocervical carcinomas with an ISMC component [6, 7]. In contrast, p40 (and/or p63) is generally more specific for true squamous differentiation. We report an ISMC case initially interpreted as SCC on biopsy and highlight a concise diagnostic approach centered on p40/p63 and mucin stains.

## Case report

**Figure 1:**
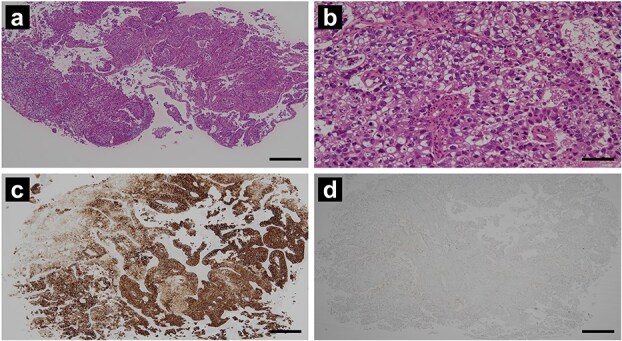
Cervical biopsy highlighting a diagnostic pitfall. (a and b) sheets and nests of atypical cells with minimal gland formation and no overt keratinization, closely mimicking non-keratinizing squamous cell carcinoma (hematoxylin and eosin (H&E)). (c) Cytokeratin 5/6 (CK5/6) shows patchy-to-diffuse positivity within tumor nests. (d) p40 is essentially negative in the tumor (with an internal positive control in non-neoplastic squamous epithelium, where present). Scale bars: (a) 500 μm; (b) 100 μm; (c) 500 μm; (d) 500 μm.

A 44-year-old premenopausal woman presented with intermittent abnormal genital bleeding. Cervical cytology suggested atypical glandular cells, and a biopsy at an outside institution was interpreted as SCC. Preoperative CT and MRI suggested stage IB2 disease, and she was referred to our hospital for further evaluation and surgical management. At our hospital, repeat cervical biopsy was initially reported as carcinoma on morphological grounds alone. Outside biopsy material, including H&E slides and p16 immunostaining, was also reviewed in parallel at our institution and was likewise interpreted conservatively as carcinoma while additional studies on the repeat biopsy were pending. Repeat cervical biopsy showed sheets and nests of atypical epithelial cells with eosinophilic to pale cytoplasm, conspicuous nuclear atypia, and only scant glandular differentiation (Fig. 1a and b). No convincing keratinization was identified. Immunohistochemistry demonstrated diffuse block-type p16 expression and focal PAX8 positivity. CK5/6 showed patchy to geographic positivity (Fig. 1c), whereas p40 and p63 were essentially negative with appropriate internal controls (Fig. 1d). Periodic acid-Schiff with and without diastase digestion (PAS/D-PAS) demonstrated diastase-resistant intracytoplasmic mucin within tumor nests, arguing against pure SCC and suggesting glandular differentiation; however, definitive subtyping was not possible on the biopsy material alone. Final classification was established only after evaluation of the hysterectomy specimen.

**Figure 2:**
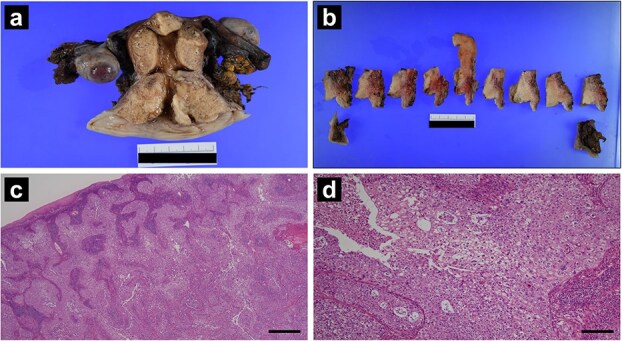
Hysterectomy specimen. (a and b) gross findings of a bulky cervical tumor. (c) Low-power view showing predominantly solid growth with a minor gland-forming component (H&E). (d) High-power view demonstrating stratified cytology within invasive nests (H&E). Scale bars: (c) 1000 μm; (d) 200 μm.

**Figure 3:**
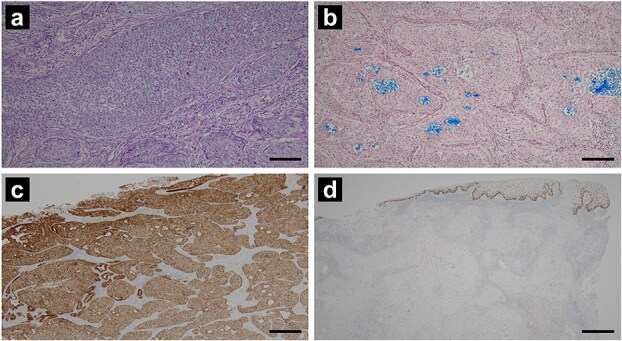
Objective demonstration of mucin and human papillomavirus-associated profile. (a) Periodic acid–Schiff with diastase digestion (D-PAS) highlights diastase-resistant intracytoplasmic mucin within invasive nests. (b) Alcian blue highlights acidic mucin in part of the tumor. (c) p16 shows diffuse block-type positivity. (d) p40 remains negative in the tumor; the surface squamous epithelium serves as an internal positive control. Scale bars: (a) 200 μm; (b) 200 μm; (c) 500 μm; (d) 500 μm.

**Figure 4:**
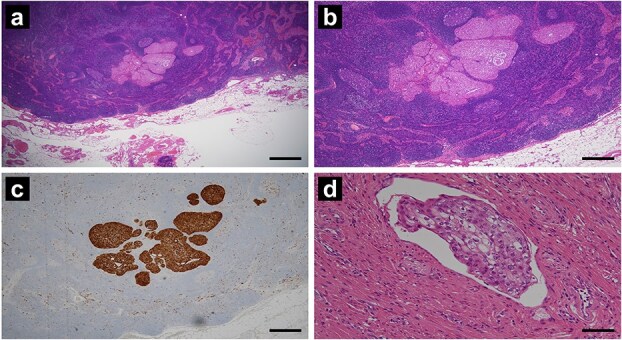
Lymphovascular invasion and nodal metastasis. (a, b) metastatic carcinoma in a regional lymph node (H&E). (c) the metastasis shows diffuse block-type p16 expression. (d) Lymphovascular tumor embolus in the cervix (H&E). Scale bars: (a) 1000 μm; (b) 500 μm; (c) 500 μm; (d) 100 μm.

The patient underwent radical hysterectomy with bilateral salpingo-oophorectomy and pelvic regional lymph node dissection. Grossly, the cervix was replaced by a bulky tumor measuring 41 × 62 mm (Fig. 2). Histologically, the tumor consisted predominantly of invasive solid nests with stratified cytology, while a minor conventional gland-forming endocervical adenocarcinoma component was present. Intracytoplasmic mucin was demonstrable within the solid nests by D-PAS and Alcian blue stains (Fig. 3a and b). The tumor showed diffuse block-type p16 expression and a wild-type p53 staining pattern; p40 remained negative (Fig. 3c and d). Lymphovascular invasion was present, and metastatic carcinoma was identified in 2 of 29 regional lymph nodes (maximum metastatic focus 2.2 mm, without extranodal extension; Fig. 4). Surgical margins and peritoneal cytology were negative. The tumor was staged as pT1b2 pN1 (International Federation of Gynecology and Obstetrics 2018 stage IIIC1). The patient received adjuvant paclitaxel plus carboplatin (six cycles). Approximately 12 months after surgery (about 5 months after completion of adjuvant chemotherapy), para-aortic nodal recurrence was detected and treated with radiotherapy, with temporary response. Programmed death-ligand 1 combined positive score was > 1; later progression raised the clinical question of immunotherapy in the setting of limited renal reserve.

## Discussion

ISMC is frequently a mixed carcinoma and may coexist with usual-type HPV-associated endocervical adenocarcinoma and/or adenosquamous carcinoma components [2, 5]. ISMC is now recognized within the spectrum of HPV-associated endocervical adenocarcinoma, reflecting its pathogenetic relationship to high-risk HPV infection. Although the present report is focused on diagnostic pathology, recognition of these HPV-associated lesions also has broader relevance in the context of cervical cancer prevention, including HPV vaccination and screening. In limited biopsies, the practical goal is to avoid misclassification as SCC when the lesion is actually an HPV-associated adenocarcinoma variant with a solid growth pattern. Our case illustrates two pitfalls: (i) nested ‘squamoid’ architecture without keratinization that makes SCC plausible on hematoxylin and eosin sections, and (ii) CK5/6 positivity despite limited specificity for squamous lineage in HPV-associated cervical carcinomas [6, 7]. In contrast, p40 (and/or p63) is typically absent or only focal/peripheral in ISMC, whereas SCC is usually diffusely positive [3, 6, 8]. Therefore, a p16 block-positive, SCC-like biopsy that is p40/p63-negative should trigger a deliberate search for intracytoplasmic mucin.

We suggest a pragmatic workflow for routine diagnostic practice. In cervical biopsies showing SCC-like solid nests without keratinization, particularly when p16 is block-positive, lineage should be tested rather than inferred: p40 (and/or p63) plus a mucin stain (D-PAS and/or Alcian blue) as a first step, with PAX8 as a supportive Mullerian marker when needed [6, 7]. If p40/p63 is negative and mucin is demonstrable within tumor nests, ISMC becomes a leading consideration; if p40/p63 is diffuse, SCC is favored. When both p40/p63 and mucin are negative, other entities should be reconsidered based on morphology and context (e.g. poorly differentiated usual-type HPV-associated adenocarcinoma without overt mucin, neuroendocrine carcinoma, or metastatic disease). This approach is useful because it relies on widely available stains applicable to small biopsy material. In the present case, the most important practical differential diagnosis was non-keratinizing squamous cell carcinoma. On limited biopsy material, both entities may show solid growth and little or no keratinization; however, diffuse squamous marker expression, especially p40/p63, favors SCC, whereas demonstrable intracytoplasmic mucin and at least focal Mullerian marker expression support an HPV-associated glandular lesion such as ISMC. Adenosquamous carcinoma may also enter the differential diagnosis, but in our case the dominant problem on biopsy was distinction from SCC in a predominantly solid lesion. ISMC may behave aggressively, with frequent lymphovascular invasion and nodal or distant metastases [4, 9, 10], consistent with our patient’s course. Accurate classification is therefore important for clinicopathological communication and future studies. Importantly, this minimum approach is feasible in routine diagnostic laboratories without extensive immunopanels or molecular testing. From a practical clinical perspective, this case also underscores the importance of close communication between pathologists and treating clinicians when biopsy findings are unusual or lineage remains uncertain. In such settings, a limited but deliberate ancillary approach may help avoid misclassification at the initial diagnostic stage and provide a more appropriate basis for subsequent multidisciplinary management.
